# Physical Activity in Adolescents following Treatment for Cancer: Influencing Factors

**DOI:** 10.1155/2013/592395

**Published:** 2013-09-18

**Authors:** Marilyn Wright, Angie Bryans, Kaylin Gray, Leah Skinner, Amanda Verhoeve

**Affiliations:** McMaster Children's Hospital, Box 2000, Hamilton, ON, Canada L8N 3Z5

## Abstract

The purpose of this study was to examine physical activity levels and influencing individual and environmental factors in a group of adolescent survivors of cancer and a comparison group. *Methods*. The study was conducted using a “mixed methods” design. Quantitative data was collected from 48 adolescent survivors of cancer and 48 comparison adolescents using the Godin Leisure-Time Exercise Questionnaire, the Fatigue Scale—Adolescents, and the Amherst Health and Activity Study—Student Survey. Qualitative data was collected in individual semistructured interviews. *Results.* Reported leisure-time physical activity total scores were not significantly different between groups. Physical activity levels were positively correlated with adult social support factors in the group of adolescent survivors of cancer, but not in the comparison group. Time was the primary barrier to physical activity in both groups. Fatigue scores were higher for the comparison but were not associated with physical activity levels in either group. The qualitative data further supported these findings. *Conclusions.* Barriers to physical activity were common between adolescent survivors of cancer and a comparative group. Increased knowledge of the motivators and barriers to physical activity may help health care providers and families provide more effective health promotion strategies to adolescent survivors of pediatric cancer.

## 1. Introduction

 The probability of survival into adulthood for children treated for cancer has increased from 30% to nearly 80% in the past 30 years [1]. Rates as high as 90% have been achieved for survivors of acute lymphoblastic leukemia [2]. However, this increase in long-term survival comes with a high risk of long-term morbidity and premature mortality from chronic diseases associated with the late effects of cancer treatments. These can include obesity, decreased bone density, poor exercise capacity, reduced muscle strength and extensibility, balance problems, fatigue, sleep problems, osteoporosis, diabetes, cardiovascular disease, pulmonary complications, and psychosocial problems [3–6]. Two-thirds of survivors of childhood cancer experience at least one late effect of cancer treatment, and as many as one-quarter experience a late effect serious enough to limit function or become life threatening [3]. 

 Participation in regular physical activity has been found to have several benefits in adult and pediatric oncology populations including amelioration of many of the noted late effects and reduced risk of some cancers [6–8]. Population levels of participation in physical activity are low in general [9]. In pediatric cancer groups, levels of physical activity decrease during therapy, and for many children, do not recover after treatment [7, 10–12]. This occurs despite the lack of contraindications to exercise in survivors of childhood cancer [7, 13]. Documented levels of physical activity vary. Some studies report less than 50% of survivors engage in regular physical activity whereas others report participation rates of over 70% [6, 7, 13]. 

An improved understanding of why some people are active and others are not is important for planning health promotion strategies and effective interventions [9]. Participation in youth is determined by dynamic interactions among the youth and their social and physical environments [9]. Several determinants for physical activity in children and adolescents have been identified. These include individual factors such as age, gender, self-efficacy, attitude towards physical activity, enjoyment and perceived benefits of physical activity; their social environment which is comprised of parents, peers, and siblings and many environmental factors such as transportation and community facilities [9, 14]. For youth treated previously for cancer, identified barriers include lack of time, pain, fatigue, a general dislike for exercise, inaccessibility to equipment and facilities, and insufficient knowledge and parental support [11, 15, 16]. Participation in physical activity is facilitated by a desire to be healthy and attractive, self-efficacy, enjoyable activities, opportunities to participate with peers and family, and activities that are perceived as “normal” [9, 16, 17]. Promotion of physical activity in survivors during adolescence is particularly important as participation in physical activity typically declines through the teen years and physical activity patterns established in adolescence can track into the adulthood [9, 14, 18].

 Previous research addressing correlates of physical activity in adolescents has been primarily quantitative in nature, and few studies have used comparison groups. Qualitative research is increasingly being used to answer complex questions confronting healthcare researchers and to reveal the meanings people attach to their experiences, thus helping to interpret social phenomena not amenable to quantitative research [19]. Mixed methods studies using both qualitative and quantitative data to explore the factors associated with physical activity in survivors of pediatric cancer and youth in general could contribute to the field of study. The purpose of this study was to study physical activity participation and related individual and environmental factors in a group of adolescent survivors of pediatric cancer and a comparison group using a mixed methods design. 

## 2. Methods

A mixed methods concurrent research design was used. Quantitative data was collected with self-report questionnaires, and qualitative data was obtained through semistructured interviews. Ethical approval was obtained from the McMaster University Research Ethics Board. A letter outlining the study purpose, methods, and assurance of confidentiality and a copy of the survey were provided to potential participants. Their completion of questionnaires was accepted as consent for involvement in the survey portion of the study. Written informed consent was obtained prior to conducting the interviews.

### 2.1. Participants

Participants were a consecutive sample of adolescents, aged 13 to 18 years, who had completed treatment for cancer during childhood or adolescence. The sample was recruited from an ambulatory oncology follow-up clinic at a tertiary care centre. An additional inclusion criterion was the ability to complete the written questionnaire. Exclusion criteria included antecedent neurological, developmental, or genetic disorders. Those with leukemia were treated using Dana Farber Cancer Institute protocols [2, 20]. All others were treated with Pediatric Oncology Group protocols if diagnosed prior to 2000 and Children's Oncology Group protocols if diagnosed after that time. A geographically similar comparison sample comprised of age- and gender-matched youth who had not been previously treated for cancer was recruited through volunteering at a local high school and subsequently through chain-referral sampling. 

### 2.2. Measures

 The quantitative data was collected using three patient-reported outcome (PRO) questionnaires that required minimal respondent burden. The Leisure Score Index of the Godin-Leisure-Time Exercise Questionnaire (GLTEQ) was used to determine physical activity participation [21]. The scale contains three questions that assess the frequency of mild, moderate, and strenuous exercise that has been done for at least 15 minutes during a typical week. A total score is calculated in arbitrary units by weighting each frequency by an estimated intensity guided by metabolic equivalents using the equation (9 × strenuous) + (5 × moderate) + (3 × light). A further question asked how often they engaged in any regular activity long enough to work up a sweat or make their heart beat rapidly in a 7-day period. Answer options were often, sometimes, or never/rarely. The questionnaire is brief, easily administered, reliable, and demonstrates concurrent validity with other tools [21]. It has been used in pediatric cancer survivors [9, 22, 23] and has been shown to correlate with accelerometer data [22].

 PRO questionnaires that addressed facilitators and barriers for physical activity in typical adolescent and oncology populations were selected to determine influencing factors [9, 11, 15–17]. The Fatigue Scale for Adolescents (FS-A) is a 13-item self-report questionnaire that was developed to measure an adolescent's perception of cancer related fatigue during the previous week [24]. Items are measured on a 5-point Likert scales. Scores range from 13 (no fatigue symptoms) to 65 (highest possible fatigue score). A cut point of 31 has been established as an indication of high fatigue. The scale has demonstrated high reliability and validity as well as the ability to detect change over time [24]. 

 An adaptation of the Amherst Health and Activity Study: Student Survey (AHAS) was administered to identify physical activity choices, sport team participation, and social support, environmental, and personal barrier factors that may impact an adolescent's ability to be physically active. It has been used in studies of adolescents and demonstrates test-retest reliability [25].

 The qualitative data was collected through individual face-to-face semistructured interviews with a subsample of the cancer survivor group who indicated an interest and availability to take part in an interview. The interview questions were developed through several focus groups with the research team to gather in-depth information and personal experiences to provide further validation of the quantitative findings through the use of open-ended questions regarding barriers and facilitators of physical activity. Interviews were completed by two members of the research team, in the participants' homes. The conversations were tape-recorded and transcribed verbatim. 

 Height and weight were extracted from clinical files for the survivors of childhood cancer. This information was collected by self-report for the comparison group. Body mass index (BMI) percentiles for age and gender were calculated [26].

### 2.3. Data Analysis

Quantitative data were analyzed using two-sample independent *t*-tests for interval-scale variables and Mann-Whitney tests for ordinal scale variables in order to determine between-group differences. Pearson correlation analyses explored associations between interval-scale variables among both the comparison and the survivors of cancer groups. Ordinal logistic regression was used for ordinal response variables. A level of *P* ≤ 0.05 was considered statistically significant. Qualitative interview transcriptions were analyzed using a directive content analysis design. Data reduction was completed through coding from a list of predetermined codes by four group members. Recurring passages that did not match a predetermined code and valuable participant quotations were discussed in a focus group consisting of the research team. The group further analyzed all apparent codes and constructed relevant themes. Data was subsequently arranged according to the theme. Quantitative and qualitative data were analysed separately and findings were combined for interpretation. 

## 3. Results

Forty-eight adolescents previously treated for cancer agreed to complete the questionnaires and two declined. Participant characteristics are outlined in Table 1. The mean age at diagnosis of cancer was 7.0 years (SD 3.4, range 1.7–14.6), and mean time off therapy was 6.9 years (SD 3.8, range 0.5–13.0). The majority were survivors of leukemia. Comparison data was collected from 48 adolescents matched for age and gender. Male and female data were combined for analyses as there were no significant group gender differences for any measures. There were no group differences between the survivors of leukemia and other types of cancer, so diagnostic groups were combined for analyses. The survivor group had significantly higher BMI percentiles for age values based on comparisons of clinic-measured values of the survivors to the self-reported values from the comparison participants.

The comparison group had higher GLTEQ total scores compared to the survivor group; however, the differences were not statistically significant. There was a significant difference between groups for the sweat/heart beat frequency question. Based on the data from the total score questions, it was calculated that 33% of survivors and 40% of the comparison group did not participate in moderate and/or vigorous physical activity most days of the week. Mean scores on the FS-A were significantly higher (denoting greater fatigue) for the comparison group. Twelve of the comparison participants scored 31 or above (the cut point for high fatigue) compared to six of the survivors. AHAS social support and environment for activity total scores did not differ between groups. Statistics for these data are outlined in Table 2. 

The AHAS personal barriers towards physical activity item scores are outlined in Table 3. There was a significant difference between the two groups in the personal barrier of “lack of time” (*P* = 0.007). Adolescents who had cancer reported that time was a barrier to physical activity less often than the comparison group. No other statistically significant differences were found between the two groups with regard to personal barriers. The activities section of the AHAS asked the respondents to indicate what physical activities they participated in. The top activities for the survivors of childhood cancer were basketball, calisthenics, cycling, running, walking for transportation, recreational swimming, and housecleaning. The comparison group indicated basketball, calisthenics, cycling, running, dancing (mainly females), walking for transportation, and housekeeping. The mean number of activities checked off for the cancer survivors was 12.0 compared to 14.1 for the comparison group. The mean number of sport teams reported by the survivor group was 1.6 versus 2.2 in the comparison group. These differences were not significant. 

### 3.1. Associations

There were no significant correlations between the survivor's years off treatment and any variable. BMI percentile for age was not associated with any variables for either group. In both groups a significant negative correlation was found between age and the GLTEQ total scores (survivors = −0.339, *P* = 0.021; comparison = −0.440, *P* = 0.002). 

There were significant correlations between GLTEQ total scores of the survivors of childhood cancer and the social support factors of adult encouragement to do physical activities or play sports (*Z* = −3.13, *P* = 0.002), having an adult telling them that physical activity was good for their health (*Z* = −3.26, *P* = 0.001), having a sibling or friend do physical activities or playing sports with them (*Z* = −2.37, *P* = 0.018), and having an adult provide transportation to a place where they could doing physical activity or sports (*Z* = −3.29, *P* = 0.001). GLTEQ was also significantly associated with the personal barriers of “lack of skills” (*Z* = −2.34, *P* = 0.019) and “the weather is too bad” (*Z* = −2.15, *P* = 0.031). In the comparison group, GLTEQ total scores were significantly associated with social support factor of having a sibling or friend do physical activities with you (*Z* = −2.06, *P* = 0.039) and the personal barriers of “lack of time” (*Z* = −3.65, *P* < 0.001), “lack of energy” (*Z* = −3.77, *P* = 0.001), “lack of skills” (*Z* = −3.51, *P* < 0.001), and “lack of knowledge on how to doing physical activities” (*Z* = −2.73, *P* = 0.006). 

### 3.2. Qualitative Data

The adolescents who were interviewed included a 17-year-old male previously treated for retinoblastoma, a 13-year-old male treated for acute lymphoblastic leukemia, and an 18-year-old female also treated for acute lymphoblastic leukemia. The qualitative data revealed the following five themes relating to motivators and barriers towards physical activity in adolescents who have had cancer: self-esteem, lack of time, normalcy, knowledge and acknowledgement regarding the health benefits of physical activity, and family influence. Quotes are listed in Table 4. 

## 4. Discussion

 Appreciation of the late effects of cancer treatment and the potential benefits of physical activity in minimizing the risk of long-term morbidity and chronic disease have made health promotion a priority in the care of childhood cancer survivors. This study used PRO and interviews to describe physical activity participation and influencing factors in adolescents following treatment for cancer and a comparison group. PRO questionnaires are important to document the impact and outcomes of cancer treatment and can contribute to improvements in clinical care and decision making [27]. The interviews provided in-depth personal perspectives. 

 There were no significant differences between the survivor and comparison groups with respect to levels of physical activity as measured by the GLTEQ total scores. This finding is similar to that of Norris et al. [23] for a group of pediatric cancer survivors and sibling controls but in contrast to the findings of Tillmann et al. [22] who reported lower mean GLTEQ total scores for survivors compared to a control group. In the current study there was a significant group difference in the reports of activity that worked up a sweat or caused a rapid heart beat suggesting that the survivors' moderate and mild exercise frequencies contributed relatively more to their GLTEQ total scores more than their strenuous exercise frequencies. 

 To achieve health benefits, a minimum threshold of physical activity must be achieved. The World Health Organization (WHO) and the Children's Oncology Group (COG) Guidelines for Diet and Physical Activity recommend that adolescents engage in moderate to vigorous physical activity for 60 minutes most days of the week [28, 29]. Although it is not possible to accurately determine this goal using the GLTEQ, it can be estimated that at least one-third of the adolescents in both groups were not meeting the WHO or COG standards based on the reported frequencies of moderate and strenuous exercise. Survivors should be assured when it is safe and advantageous to exercise at these levels.

 Participation in physical activity is determined by dynamic interactions among individual factors and social and physical environments [9]. An understanding of these factors can inform the development of multilevel interventions that offer the best chance for success [9]. The individual factor of age was inversely correlated with the GLTEQ total scores in both groups, a finding consistent with other studies [9, 14]. However, neither gender nor BMI-for-age were associated with any variables in this study.

Surprisingly, the comparison group reported significantly higher fatigue scores relative to the survivor group and had twice the number of respondents reporting an FS-A score above the cut point for high fatigue. It was possible that the survivors had a different benchmark for fatigue after experiencing treatment-related fatigue on therapy and rated their current fatigue to be less comparatively. FS-A scores were not associated with GLTEQ total scores for either group although “lack of energy” was a relatively highly rated barrier to physical activity. Other research has noted fatigue to be a barrier to physical activity in youth surviving cancer [15–17]. 

Motivation for participation in physical activity is an intrinsic personal factor, often based on other personal and environmental factors. The survivors' narrative data suggested that this subsample understood the importance of and were interested in regaining the strength and endurance lost during treatment, maintaining a healthy weight, and enjoying overall health now and in the future. These views could have been influenced by education about the benefits of and guidelines for physical activity from health professionals [29]. The variability in physical activity data suggests that some survivors do not have or act on these motivators. The interviews also revealed a desire to achieve normalcy by regaining their previous participation in activities and keeping up with their peers. The between-group similarities in their PRO physical activity choices and sport team participation were an encouraging indication of achieving this goal. 

 The highest rated AHAS personal barriers to physical activity for both groups were “lack of time,” “lack of self-discipline,” and “lack of energy,” however these were rated “often” or “very often” by less than 20% of participants. “Lack of time” was the only personal barrier significantly different between groups (greater for comparison group). GLTEQ total scores were associated with the barriers of “lack of skills” in both groups as well as “lack of time,” “lack of knowledge,” and “lack of energy” in the comparison group. The latter barrier could be related to the comparison group's relatively high fatigue scores. Arroyave et al. reported “being too tired,” “too busy,” and “not belonging to a gym” as barriers to physical activity in childhood cancer survivors [16] however there was no control group to provide perspective. Takken et al. suggested schoolwork or social activities could be barriers to physical activity [15].

 The Environment for Activity AHAS scores suggested both groups lived in physical environments that provided access to exercise equipment, facilities, and safe neighbourhoods. The social support AHAS scores demonstrated overall moderate social support for exercise. Encouragement, espousing health benefits, providing transportation, and having a sibling or friend to do physical activities with positively correlated with GLTEQ total scores. The sibling or friend factor also showed a positive correlation for the comparison group. The qualitative data reflected the supportive roles of families but also indicated that some parents could be restrictive.

 The importance of families as facilitators of physical activity in this study is supported by Norris et al. who noted that a link exists between social support and physical activity and that family is the most important social influence in the promotion of healthy behaviours [23]. Therefore family education on the importance of physical activity and strategies to integrate physical activity into the busy day-to-day lives of these children both during and following treatment should be a standard of care [30]. Recognition of inactive survivors those and maintaining physical activity participation into older adolescence and adulthood is essential. 

 The findings of this study can be generalized to groups with similar treatment experiences and living environments. Study limitations include the possibility of volunteer bias and small sample size for the interviews; however, saturation was becoming evident after only three interviews. It was difficult to compare the groups BMI percentiles as the survivors' weights and heights were measured in a clinic whereas the comparison group were self-reported. The comparison group's scores are suspect given the national estimates of overweight and obesity are 27.9% [31]. The cross-sectional methodology assesses statistical association but does not allow causal inference [9]. Longitudinal observational research to identify life course trajectories and determinants of physical activity and interventional studies to evaluate health promotion strategies are necessary to further the understanding of physical activity in survivors of childhood cancer. 

## Figures and Tables

**Table 1:** Participant characteristics.

Characteristic	Survivor *N* = 48	Comparison *N* = 48	*P* value
Age in years—mean (SD)	16.0 (2.1)	15.8 (1.7)	NS
% Male	60.5	62.5	NS
BMI percentile—mean (SD)	60.5 (27.8)	44.9 (25.2)	0.005
(i) % Under weight	2.1	4.2	
(ii) % Healthy	74.5	87.5	
(iii) % Overweight	14.9	6.2	
(iv) % Obese	8.5	2.1	
Diagnosis			
(i) % Leukemia	66.6		
(ii) % Solid tumour	12.5		
(iii) % Lymphoma	18.7		
(iv) % CNS tumour	2.1		

NS: not significant.

**Table 2:** Mean (standard deviation) values for the GLTEQ total scores, FS-A, and Social Support and Environment for Activity sections of the AHAS and frequencies for the GLTEQ sweat/heart beat question.

	Survivor	Comparison	*P* value
GLTEQ total	60.0 (32.8)	65.5 (32.4)	NS
GLTEQ sweat/heart beat			0.015
(i) # Never	3	1	
(ii) # Sometimes	28	18	
(iii) # Often	16	27	
Fatigue	10.1 (7.4)	14.0 (8.3)	0.017
Social support	19.1 (5.7)	19.1 (6.2)	NS
Environment for activity	10.2 (1.8)	10.5 (1.5)	NS

**Table 3:** Reported personal barriers to physical activity % represents the proportion reporting “often” and “very often” and between group differences.

Personal barrier	Survivor		Comparison		*P *value
	%	*M* (SD)	%	*M* (SD)	
Lack of energy	19.2	1.4 (1.1)	17.0	1.5 (1.2)	NS
Lack of self-discipline	19.2	1.3 (1.2)	25.5	1.5 (1.2)	NS
Lack of time	17.0	1.5 (1.1)	46.8	2.4 (1.1)	0.007
Lack of skills	10.6	1.0 (1.1)	8.5	0.9 (1.0)	NS
No one to do physical activity with	10.4	1.1 (1.2)	12.8	1.3 (1.2)	NS
The weather is bad	8.5	1.2 (0.9)	4.3	1.3 (1.0)	NS
Self-conscious about my looks when exercising	6.4	0.8 (0.9)	8.5	0.9 (1.1)	NS
Lack of knowledge about how to be physically active	4.2	0.6 (0.8)	6.4	0.7 (0.8)	NS
I am too overweight	2.1	0.3 (0.8)	0	0.2 (0.5)	NS
Physical activity is boring	2.1	0.8 (0.9)	2.1	0.8 (1.0)	NS
Friends tease me during exercise or sports	2.1	0.3 (0.6)	0	0.4 (0.6)	NS
Someone told me not to exercise	2.1	0.1 (0.3)	0	0.1 (0.4)	NS

NS: not significant.

**Table 4:** Qualitative quotes matching the four main themes.

Theme	Quotes
Self-esteem	“…*makes me feel better about myself and makes me feel good after I'm done*”
	*“[physical activity] makes you feel better about yourself” *
	*“I think [physical activity] gives me a brighter outlook…like wanting me to be better…” *

Lack of time	*“sometimes school work gets in the way” “…the workload and the stress…” *
	*“…if you have homework going on or if you have a social event…it's hard to be physically active…” *
	*“it's not so much the place, it is the time” *

Normalcy	*“…I've been pretty good at keeping up with the others…I feel like I'm kinda, you know, on par with everyone else…like keeping it up and doing just as well as the others…” *
	*“…I still lived normally, like playing with cars and stuff…” *
	*“ I do not think that treatment changed [my exercise behaviours]. …I just do different things.” *
	*“I would say just go outside and slowly start building up again. If there was something you liked to do before treatment, just try and get back to it. Slowly get back into the groove of things”. *

Knowledge and acknowledgement of the health benefits of physical activity	*“…build muscle strength back…” *
	*“…like avoiding obesity and everything…” *
	*“…live a long and healthy and happy life.” *
	*“…first build up your muscle…about stamina….it will make you healthier in the long run.” *
	“*I know I am getting stronger as I do it and that is a good feeling*”
	“…*your priorities change*….*now the time that used to go into certain things changed and* *now go into exercising and dance and stuff like that*…”.

Family influence	“[*parents*]…*just reminding me*…*if I haven't been on the treadmill in a while*…”
	“[*Mom would say*] *go outside and play*.”
	“*I think they were sometimes more the barrier*…*but I've been working on that*…*not* *so much anymore*…*They just wanted me to focus on studies rather than myself*”.
